# Transitory versus Persistent Effects of Connectivity in Environmentally Homogeneous Metacommunities

**DOI:** 10.1371/journal.pone.0044555

**Published:** 2012-08-30

**Authors:** Romana Limberger, Stephen A. Wickham

**Affiliations:** 1 Department of Biology, McGill University, Montreal, Canada; 2 Department of Organismic Biology, University of Salzburg, Salzburg, Austria; CNRS, University of Montpellier II, France

## Abstract

While the effect of habitat connectivity on local and regional diversity has been analysed in a number of studies, time-dependent dynamics in metacommunities have received comparatively little consideration. When local patches of a metacommunity are identical in environmental conditions but differ in initial community composition, dispersal among patches may result in homogenization of local communities. In a microcosm experiment with benthic ciliates, we tested the hypothesis that the effect of connectivity on diversity is time-dependent and only transitory, with the degree of connectivity affecting the time to homogenization but not the final outcome. Six microcosms were connected to a metacommunity with one of three levels of connectivity. The six patches differed in initial community composition but were identical in environmental conditions. We found a time-dependent and transitory effect of connectivity on local and regional richness and on local Shannon diversity, while Bray-Curtis dissimilarity and regional Shannon diversity were persistently affected by connectivity. Local richness increased and regional richness decreased with connectivity during the initial phase of the experiment but soon converged to similar values in all three connectivity treatments. Local Shannon diversity was unimodally related to time, with maximum diversity reached sooner with high than with medium or low connectivity. Eventually, however, local diversity converged to similar values irrespective of connectivity. At the regional scale, Shannon diversity was persistently lower with high than with low connectivity. While initial differences in community composition vanished with medium and high connectivity, they were maintained with low connectivity resulting in persistently high beta and regional diversity. The effect of connectivity on ciliate community composition translated down to the algal resource, as stronger dominance of the superior competitor with high and medium connectivity resulted in stronger depletion of the resource.

## Introduction

Understanding the mechanisms that underlie patterns of biodiversity is a fundamental challenge in ecology. In recent years, the metacommunity concept has added new insight into mechanisms behind diversity by considering both local and regional processes and their interaction in structuring local communities [1], with a metacommunity being defined as a set of local communities linked by dispersal of multiple potentially interacting species [2].

In metacommunities structured by competition, a number of different mechanisms can affect diversity, depending on whether metacommunities are spatially homogeneous or heterogeneous [1], [3]. In spatially homogeneous metacommunities, patch dynamics and neutral dynamics can influence diversity [1], while in spatially heterogeneous metacommunities, species are able to coexist on the scale of the region by specialization on the different habitat types [1], [3]. When dispersal between these heterogeneous patches is sufficiently strong, even local coexistence is possible through continuous migration of species from their preferred habitats (sources) to sink communities where they are inferior competitors [1], [3]. A high degree of connectivity, however, has been predicted to result in homogenization of local communities and dominance of the regionally superior competitor in all local communities. For spatially heterogeneous metacommunities, modelling thus predicts a unimodal relationship between connectivity and local diversity and a negative effect of connectivity on beta and regional diversity [4].

Patterns similar to those predicted for spatially heterogeneous metacommunities have been found in environmentally homogeneous metacommunities when patches initially differed in community composition [5], [6]. In such metacommunities, homogeneous in environment but heterogeneous in community history, connectivity enables species to overcome dispersal limitation, thereby increasing local diversity through immigration of species but decreasing beta and regional diversity through homogenization of communities. An important difference of environmentally heterogeneous metacommunities, however, is that local patches remain dissimilar in environmental conditions while initial heterogeneity in community composition in environmentally homogeneous metacommunities is not necessarily maintained over time. In environmentally homogeneous metacommunities, even low dispersal may result in homogenization of communities, and in that case, the effect of connectivity would be only transitory and strongly time-dependent. However, few studies have analysed time-dependent dynamics in metacommunities. In a modelling approach with random colonization from a regional species pool, diversity of a local community was unimodally related to time [7]. Local species richness initially increased due to transitory coexistence of species and then decreased due to competitive effects. Time-dependent effects of connectivity have also been found by empirical studies [6], [8]. In an experiment comparing connected and unconnected metacommunities, the positive effect of connectivity through immigration of dispersal-limited species was soon offset by immigration of a predator [8], resulting in a transitory effect of connectivity. If, however, only high dispersal rates result in homogenization, but initial differences in community composition remain with low dispersal, then a persistent effect of connectivity would be possible despite absence of environmental heterogeneity. Increasing importance of community history with increasing isolation would be the mechanism driving diversity patterns in such metacommunities [9], [10].

Effects of connectivity on ecosystem functioning is also a topic that has received comparatively little consideration in empirical studies. Metacommunity experiments analysing ecosystem functions found connectivity to affect biomass production [5], ecosystem stability [11], and dynamics of carbon and nitrogen [12]. Resource depletion is another ecosystem process that might be affected by dispersal-related changes in diversity, given empirical evidence for increasing resource use efficiency with increasing diversity [13], [14]. Experiments with aquatic mesocosms that considered more than one trophic level found effects of connectivity on consumer communities to translate down to the resource community [15], [16]. In most microcosm experiments, however, resource dynamics in metacommunities have received no attention.

Here we present a microcosm experiment testing for interactive effects of connectivity and time on the diversity of a ciliate model community and on the dynamics of their resources. The five ciliate species used in the experiment competed for a benthic diatom and for bacteria. In addition, one species pair also interacted through omnivory. A metacommunity consisted of six microcosms, connected by active dispersal according to one of three levels of connectivity. The six patches of a metacommunity had identical environmental conditions but differed in initial species composition. In contrast to many earlier metacommunity experiments, we frequently sampled the microcosms to obtain a detailed time series of ciliates and their resources. Rather than comparing unconnected with connected metacommunities, we compared three connected treatments with large differences in the degree of connectivity. We predicted that the effect of connectivity on local, regional, and beta diversity would depend on time and be only transitory. Local diversity should be unimodally related to time, initially increasing due to immigration of dispersal-limited species, and then decreasing due to immigration and dominance of strong competitors. The speed of diversity increase and decrease should be faster the higher the degree of connectivity, resulting in strong time-dependency of the connectivity-diversity relationship. Regional diversity and beta diversity (measured as Bray-Curtis dissimilarity) should decrease with time due to homogenization of initially different communities, with the degree of connectivity affecting only the speed of diversity decline but not the final outcome. Alternatively, initial differences in community history could leave a stronger imprint on community structure in weakly connected metacommunities, resulting in persistently high local, beta and regional diversity with low connectivity. The second aim of this study was to analyse effects of connectivity on the resource community. We expected time-dependent effects of connectivity on the ciliate community to translate into time-dependent effects on the resource community.

## Methods

### Ethics statement

No specific permits were required for the described study. The organisms used for the experiments were isolated from locations that are open to the public and are not protected in any way. The study did not involve endangered or protected species.

### Test organisms

We tested our hypotheses using monoclonal cultures of five benthic ciliate species (*Stylonychia pustulata, Onychodromopsis flexilis, Frontonia angusta, Paramecium caudatum, Urostyla grandis*; henceforth abbreviated by genus names). These species were isolated from freshwater habitats around the city of Salzburg, Austria. The ciliates were cultured in 0.2 µm-filtered pond water and fed on the benthic diatom *Navicula pelliculosa* obtained from the culture collection at Göttingen (SAG). Algal and ciliate cultures were non-axenic and contained a variety of bacteria. When grown in mixed cultures, the largest of the five species, *Urostyla*, showed predatory effects and fed upon the smallest species, *Stylonychia*, driving it to extinction. From previous experiments, data on colonization and competitive ability is available for four of the five species [17]. Since data on these traits was missing for *Urostyla*, we measured its colonization ability and found it to need 2.7 days to colonize a basin separated by 10 cm from the source community. It was thus an intermediate colonizer, slower than *Stylonychia* and *Paramecium*, but faster than *Onychodromopsis* and *Frontonia*.

Partitioning colonization ability into its two components, dispersal and growth, can give further insight into mechanisms behind patterns [18], [19]. Data on dispersal and growth rates is available for some of the species (*Stylonychia*, *Paramecium*, *Frontonia*) [17]. We also measured these two traits for *Urostyla* and found it to have a comparatively low growth rate (0.33 ind ind^−1^ day^−1^) and an intermediate dispersal rate (0.018 ind ind^−1^ day^−1^), measured as in Limberger and Wickham [17].

### Experimental design

**Figure 1:**
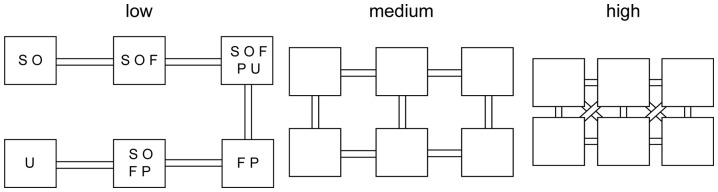
Experimental design and initial species distribution. Each metacommunity consisted of six microcosms that were connected according to one of three connectivity treatments, differing in the number and length of connections and in the amount of time the connections were open (low connectivity: 1 or 2 connections per basin, 15 cm length, open 4 h per week; medium connectivity: 2 or 3 connections per basin, 10 cm length, open 48 h per week; and high connectivity: 3 or 5 connections per basin, 4 cm length, 8 cm diagonales, always open). The initial species distribution was randomized and the same for all treatments, shown here in the treatment with low connectivity. S  =  *Stylonychia*, O  =  *Onychodromopsis*, F  =  *Frontonia*, P  =  *Paramecium*, and U  =  *Urostyla.*

We used small plexiglass basins (12×12×8 cm) filled with 300 ml of 0.2 µm-filtered pond water as microcosms. To simulate a benthic system, we placed 25 ceramic tiles (2.27×2.27×0.5 cm) covered with a biofilm of the benthic diatom and associated bacteria into each basin. The tiles had been incubated with bacillariophycean medium and an inoculum of *Navicula pelliculosa* four days prior to the start of the experiment and then transferred into the microcosms. We connected six basins to a metacommunity with silicon tubing of 0.5 cm inner diameter and compared three levels of connectivity (Fig. 1): low connectivity (1.7 connections per basin, tubing of 15 cm length, open for 4 hours per week), medium connectivity (2.3 connections per basin, tubing of 10 cm length, open for 48 hours per week), high connectivity (3.7 connections per basin, tubing of 4 cm length and diagonales of 8 cm, always open). When connections were closed, we blocked them in the middle using tube clamps. The treatments thus differed in the number of connections per basin, in the length of connections, and in the amount of time the connections were open. The aim of this setup was to create metacommunities with large differences in their degree of connectivity. However, with this design it was not possible to disentangle effects of the different modes of connectivity, in that connection distance was inversely related to connection duration. All treatments were replicated three times and conducted at 20°C with a light: day cycle of 12∶12 hours.

Initially, we compared the three connected treatments with large basins of the same volume as six small basins, hence a treatment with maximum possible connectivity. However, the ciliates grew very poorly in these large basins, and thus we implemented a treatment with six unconnected basins and hence minimum possible connectivity. The treatment without connections was thus conducted after the three connected treatments. Since we cannot fully exclude that timing of the experiment influenced the results, we show data from the unconnected treatment only as a supplemental file (Fig. S1).

At the beginning of the experiment, the five ciliate species were added to the six basins according to a random distribution (Fig. 1). First, a random number between 1 and 5 was drawn for each of the six microcosms to determine the species number for each patch. Then the species identity was determined, again using random numbers. The same initial distribution was used for all treatments and replicates, and the same total ciliate biovolume (77×10^6^ µm^3^) was introduced into each basin. Thus, while the initial species composition was chosen randomly, there was no variation in their assignment – each treatment received exactly the same set of species combinations. As a result, the species combination was treated as a fixed factor in the statistical analysis. To ensure that the microcosms initially differed only in ciliate but not in bacterial communities, we filtered a part of each of the five ciliate cultures through a 5 µm filter and added the filtrates to each of the basins. Thus, bacteria associated with the ciliate cultures were introduced into each basin.

### Sampling

Over a period of 8 weeks, we weekly sampled each basin for abundances of ciliates and resources. Before sampling, we blocked the connections with tube clamps to avoid the creation of currents and passive transfer of organisms. We then removed three tiles from each basin with the help of a plexiglass sampler. One tile fitted tightly into the sampler, allowing the removal of a tile and the water column above it. The removed tiles were replaced by three resource-covered tiles, and the removed water was replaced by filtered pond water. To ensure algal growth, the pond water was enriched with the nutrients that were also used to prepare the bacillariophycean medium. The biofilm on the sampled tiles was scraped off with a razor blade, after carefully rinsing the tiles with filtered pond water. We then merged the withdrawn water with the rinsing water and the biofilm to give a 75 ml sample volume. Depending on the ciliates' abundances, up to 3 ml were live-counted under a dissecting microcope. Due to comparatively low abundances of *Urostyla*, up to 20 ml were counted for this species, particularly during the initial phase of the experiment.

Algal abundances were measured fluorometrically and fluorescence values were transformed to abundance values after calibrating the fluorometer with samples of known algal abundance. Bacteria were quantified by flow cytometry. A subsample was fixed with glutardialdeyhde (4% final concentration) and sonicated to disaggregate clumps of algae and bacteria. The samples were shock-frozen in liquid nitrogen and stored at −70°C until quantification by cytometry. Following Marie et al. [20], samples were stained with SYBR Green I (Molecular Probes) and measured on a FacsCanto II flow cytometer (Becton-Dickinson) equipped with an argon laser (488 nm). To differentiate small and medium-sized bacteria, cyanobacteria, and diatoms, the cells' forward scatter, side scatter, SYBR Green-induced green fluorescence, phycoerythrin-induced orange fluorescence and chlorophyll-induced red fluorescence were measured. Data acquisition and analysis was performed with FACSDiva Software (Becton-Dickinson).

Since the basins became contaminated with microflagellates, we quantified flagellates in all our samples. Therefore, a subsample was fixed with glutardialdehyde (2% final concentration), and then gently sonicated. A DAPI-stained subsample was filtered onto a black polycarbonate membrane filter (0.2 µm pore size; Nuclepore). Flagellates and bacteria too large to be quantified by cytometry (>10 µm length) were counted by epifluorescence microscopy in 50 randomly selected fields.

### Data analysis

We calculated two measures of diversity: species richness and the Shannon-Wiener index [21], with the latter taking both richness and dominance structure of the community into account. Throughout the text, we use the term “richness” when meaning species richness, and the terms “Shannon diversity” or “diversity” when meaning the Shannon-Wiener index. We computed these measures of diversity at both the local and the regional scale. Local or alpha diversity was species richness and Shannon diversity, respectively, computed for each local community and then averaged over the six communities of a metacommunity. Regional or gamma diversity was based on mean abundances in the whole metacommunity when computing regional Shannon diversity, and on total species number in the metacommunity when computing regional species richness. To measure beta diversity, we calculated Bray-Curtis distances between the six communities of a metacommunity.

We used repeated-measures (rm) ANOVAs with time as within-subject factor and connectivity as between-subject factor to test for effects of connectivity and time on diversity, species richness, Bray-Curtis distance, ciliate and resource abundances. Tukey's post-hoc tests served to identify treatments with significant differences. When the assumption of sphericity was violated, a Greenhouse-Geisser correction was used. Dissimilarity values were arcsine square-root transformed, since the Bray-Curtis distance is confined to the range from 0 to 1. Abundances of ciliates, algae, bacteria and flagellates were log_10_-transformed prior to analyses.

To test for an effect of initial species composition on final local diversity and for a possible interaction with connectivity, we computed another repeated-measures ANOVA, with the six communities as spatial repeated measures. In this rm-ANOVA, initial community composition was the within-subject factor and connectivity the between-subject factor. We thus took account of the fact that the six local communities of a metacommunity were not independent from one another. Statistical analyses were conducted with PASW 18.0 for Windows.

## Results

### Diversity

**Figure 2:**
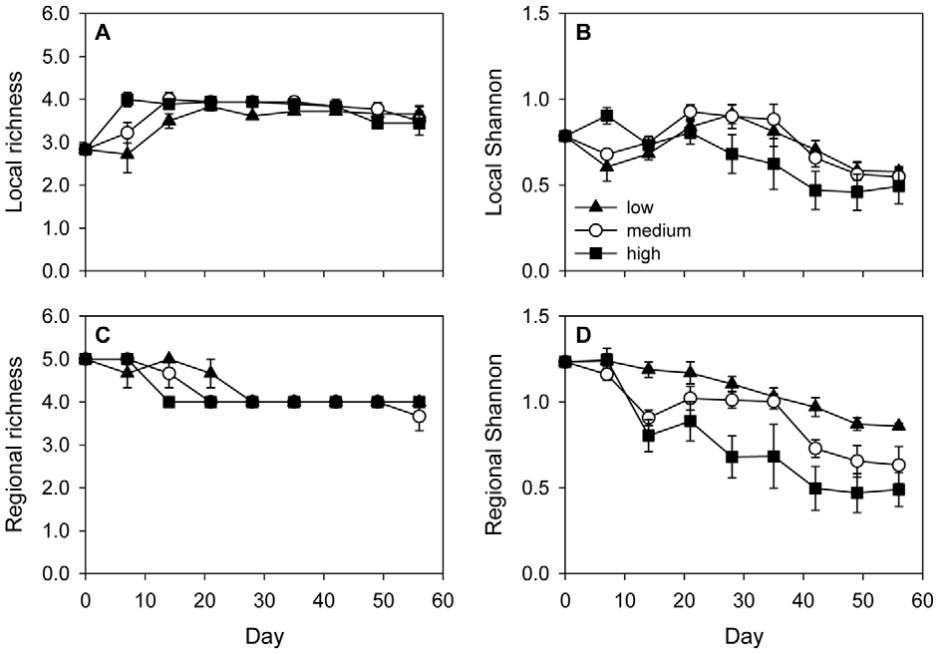
Local and regional diversity with low, medium, and high connectivity. The measures of diversity used were species richness (A, C) and the Shannon-Wiener index (B, D), computed at the local (A, B) and regional scale (C, D), respectively. Values are means ± SE, n = 3.

Time strongly affected both local and regional diversity and in some cases interacted with connectivity in structuring diversity (Table 1, Fig. 2). When averaged over time, local species richness was unaffected by connectivity (Table 1, Fig. 2A). The time to reach maximum local richness, however, clearly depended on the level of connectivity, with a faster increase in species richness the higher the degree of connectivity (Fig. 2A). Similarly, local Shannon diversity increased faster with high than with medium or low connectivity (Fig. 2B). Local diversity showed a pronounced unimodal relationship with time in medium and low connectivity treatments, while consistently declining with high connectivity after the initial increase. Eventually, local diversity in all three treatments converged. Across time, local Shannon diversity was unaffected by connectivity and the interaction with time was only marginally significant (Table 1, Fig. 2B).

**Table 1:** Results of one-way rm-ANOVAs testing for effects of time and connectivity on measures of local and regional diversity, on beta diversity expressed as Bray-Curtis distance, and on mean local abundances of the five ciliate species.

	time		time × connectivity		connectivity	
	F_7,42_	P	F_14,42_	P	F_2,6_	P
Local richness	4.822	**0.030**	2.526	0.098	2.830	0.136
Local Shannon	12.991	**0.001**	2.709	0.080	1.008	0.419
Regional richness	20.593	**<0.001**	3.370	**0.033**	2.600	0.154
Regional Shannon	27.946	**<0.001**	2.002	0.126	8.087	**0.020**
Bray-Curtis	16.575	**0.001**	0.630	0.636	10.272	**0.012**
*Stylonychia*	131.185	**<0.001**	4.934	**0.018**	4.066	0.077
*Frontonia*	11.632	**0.002**	1.186	0.367	17.133	**0.003**
*Onychodromopsis*	44.303	**<0.001**	1.453	0.274	3.617	0.093
*Paramecium*	82.520	**<0.001**	3.728	**0.028**	0.035	0.966
*Urostyla*	51.407	**<0.001**	3.285	**0.047**	1.100	0.392

Ciliate abundances were log_10_-transformed prior to analyses. When the assumption of sphericity was violated, a Greenhouse-Geisser correction was used. P-values <0.05 are in bold; n = 3.

**Figure 3:**
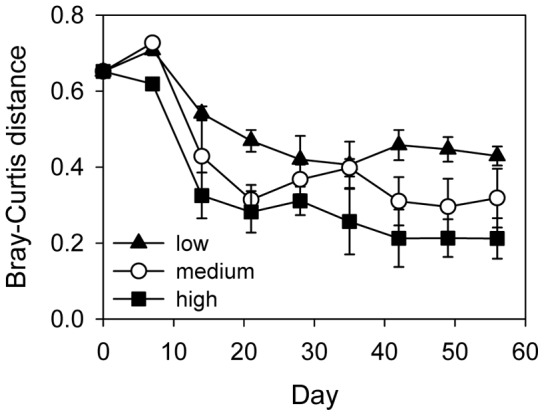
Beta diversity in the three connectivity treatments. Dissimilarity of local communities in a metacommunity was calculated as Bray-Curtis distance. Values are means ± SE, n = 3.

At the regional scale, species richness was unaffected by connectivity when averaged over time. However, the effect of connectivity was time-dependent, with regional richness declining faster the higher the degree of connectivity (Table 1, Fig. 2C). Regional Shannon diversity decreased with time, and it did so to a faster extent the higher the degree of connectivity (Table 1, Fig. 2D). Over nearly the entire course of the experiment, regional Shannon diversity was significantly lower with high than with low connectivity. With medium connectivity, however, regional Shannon diversity was comparatively high during the middle period of the experiment before declining towards the end of the experiment. A similar pattern emerged for beta diversity, measured as the Bray-Curtis distance (Table 1, Fig. 3). This was persistently lower with high than with low connectivity.

### Species' abundances

The five ciliate species were differently affected by connectivity (Table 1, Fig. S2). *Stylonychia*, the smallest species, went extinct in all treatments due to predation by *Urostyla* (Table 1, Fig. S2A). Time to extinction depended on the level of connectivity, with high and medium connectivity resulting in especially fast extinction. Mean local abundance of *Frontonia* was unimodally related with time and strongly affected by the degree of connectivity, reaching higher abundances with low and medium than with high connectivity (Table 1, Fig. S2B). *Onychodromopsis* consistently increased with time and was nearly significantly more abundant with high than with low connectivity (Table 1, Fig. S2C), while *Paramecium* and *Urostyla* were largely unaffected by connectivity (Table 1, Fig. S2D, E).

Patterns of final patch occupancy reflected those of species' abundances (Table 2). *Paramecium* was present in all patches, irrespective of connectivity. Similarly, *Urostyla* was absent from only one patch in one of the metacommunities with medium connectivity. Patch occupancy of *Onychodromopsis* was higher the higher the degree of connectivity, while the reverse was true for *Frontonia*. However, over the course of the experiment, *Frontonia* had built up populations in all of the patches, while *Onychodromopsis* had never built up populations in those patches from which it was absent in the end.

**Table 2:** Final patch occupancy of the five species in the three connectivity treatments.

Species	low	medium	high
*Stylonychia*	0	0	0
*Frontonia*	14	11	8
*Onychodromopsis*	16	17	18
*Paramecium*	18	18	18
*Urostyla*	18	17	18

**Figure 4:**
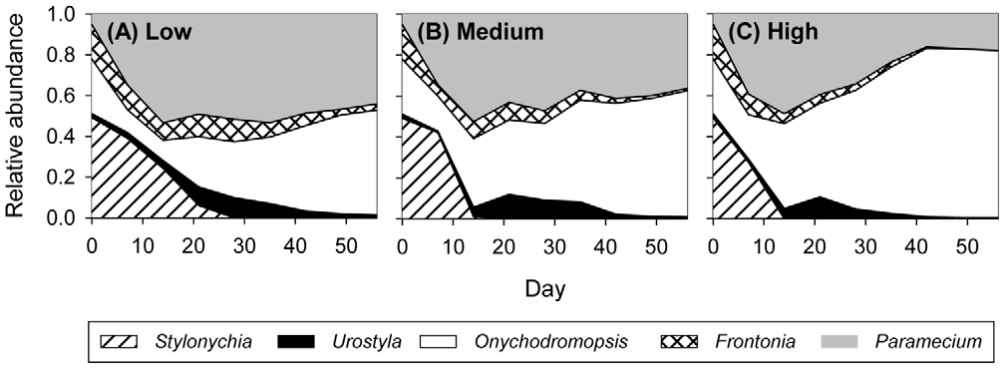
Relative metacommunity abundances of the five ciliate species. (A) low connectivity treatment, (B) medium connectivity treatment, and (C) high connectivity treatment.

Species-specific responses to connectivity translated into differences in community composition (Fig. 4). The most important species in terms of relative metacommunity abundance were *Onychodromopsis* and *Paramecium*. While these two species maintained approximately equal relative abundances in the low connectivity treatment (Fig. 4A), *Onychodromopsis* reached very strong dominance with high connectivity (Fig. 4C).

### Effect of initial species composition

**Figure 5:**
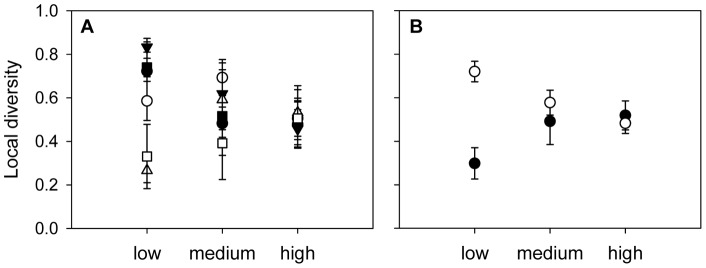
Effect of initial species combination on final local diversity. Values were averaged (A) over communities with the same initial species combination (means ± SE, n = 3) and (B) over communities with and without initial presence of *Onychodromopsis* (means ± SE, n_with_  = 12, n_without_  = 6), respectively. Symbols depict (A) the six initial species combinations (SO: closed circles, SOF: open circles, SOFPU: closed triangles, U: open triangles, SOFP: closed squares, FP: open squares; species abbreviations as in Fig. 1) and (B) communities with initial presence of *Onychodromopsis* (open circles) and without initial presence of *Onychodromopsis* (closed circles).

Whether initial species composition affected final local diversity of the communities depended on the level of connectivity (rm-ANOVA: initial species composition: F_5,30_  = 2.287, P = 0.071; connectivity: F_2,6_  = 0.385, P = 0.696; species composition × connectivity: F_10,30_  = 2.264, P = 0.041). While final local diversity was very similar in all six communities of a metacommunity with high connectivity, differences remained with low connectivity (Fig. 5A). Here, final local diversity was particularly low in those communities where the superior competitor *Onychodromopsis* was initially absent and particularly high in communities with initial presence of *Onychodromopsis* (Fig. 5B).

### Resources

**Figure 6:**
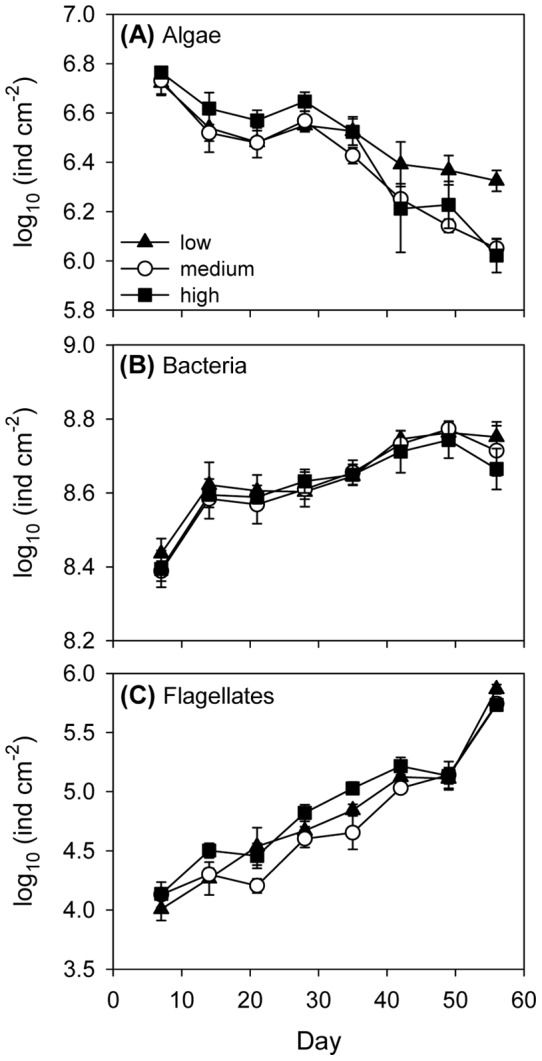
Resource abundances in the three connectivity treatments. (A) algae, (B) bacteria, and (C) flagellates, means ± SE, n = 3.

Averaged over the entire experiment, algal abundances were unaffected by connectivity (rm-ANOVA: F_2,6_  = 1.184, P = 0.369; Fig. 6A). However, during the last weeks of the experiment, algal abundances declined with medium and high connectivity, resulting in final algal abundance to be lower with medium and high connectivity than with low connectivity (ANOVA: F_2,6_  = 10.550, P = 0.011). Correlations between final algal and ciliate abundances were computed to detect a possible reason for the treatment-specific differences in algal abundance. Algal abundance was negatively correlated with total ciliate abundance (Pearson's r = −0.347, P = 0.010, n = 54). Among the five ciliate species, only *Onychodromopsis* was marginally significantly correlated with final algal abundance (Pearson's r = −0.256, P = 0.060, n = 54). However, abundance of *Onychodromopsis* and total ciliate abundance were strongly and positively correlated (Pearson's r = 0.759, P<0.001, n = 54).

Bacterial abundances increased over the course of the experiment but were unaffected by connectivity, both averaged over the experiment and dependent on the time point (time: F_7,42_  = 26.497, P<0.001, time × connectivity: F_14,42_  = 0.283, P = 0.929, connectivity: F_2,6_  = 0.406, P = 0.683; Fig. 6B). Flagellates increased from beginning to end of the experiment but were unaffected by connectivity (time: F_7,42_  = 158.701, P<0.001, time × connectivity: F_14,42_  = 1.732, P<0.168, connectivity: F_2,6_  = 2.728, P<0.144; Fig. 6C).

## Discussion

In our environmentally homogeneous metacommunities, we found both transitory and persistent effects of connectivity on diversity, depending on the spatial scale of observation and the measure of diversity used. We confirmed our hypothesis of time-dependent and transitory effects of connectivity for the local scale and for regional richness, but not for beta and regional Shannon diversity (Fig. 2, 3). Local richness increased and regional richness decreased with increasing connectivity during the initial phase of the experiment. Both local and regional richness soon converged to similar values in all connectivity treatments, resulting in the effect of connectivity being time-dependent and transitory. Local Shannon diversity was unimodally related to time, first increasing due to immigration of dispersal-limited species, and then declining due to increasing competitive effects. This sequence was faster with high connectivity than with medium or low connectivity. Eventually, however, average local diversity converged to similarly low values in all three connectivity treatments. The effect of connectivity on local Shannon diversity was thus initially positive, then slightly negative, and finally nonsignificant. While effects of connectivity on local and regional richness and on local diversity were only transitory, connectivity had persistent effects on regional Shannon diversity and on beta diversity. Both were higher with low than with high connectivity over the entire course of the experiment, as initial differences in community composition were maintained in weakly connected metacommunities but vanished with high and medium connectivity (Fig. 5A).

The mechanisms behind these patterns can be elucidated using information on species' traits and abundance data. A previous study on colonization and competitive abilities of our test organisms showed that *Onychodromopsis* was by far the strongest competitor in the community, but was only intermediate in its colonization ability [17]. The present study adds that *Onychodromopsis* had severe difficulties in invading already established communities when connectivity was low. In highly connected metacommunities, *Onychodromopsis* gained relative abundances of around 80% in all local communities irrespective of initial presence or absence, while in weakly connected metacommunities it reached around 65% and 1% in local communities with and without its initial presence, respectively (Fig. S3A). Both local and regional dominance of *Onychodromopsis* were thus considerably lower with low connectivity. Local communities that resisted invasion of *Onychodromopsis* became strongly dominated by *Paramecium* (Fig. S3B). In contrast to *Onychodromopsis*, *Paramecium* had a high colonization ability, based on an intermediate growth rate and a very high dispersal rate [17]. These traits enabled *Paramecium* to quickly reach all basins even with low connectivity, although the species was initially absent from more patches than *Onychodromopsis*.

Presence of these two alternative community states in weakly connected metacommunities resulted in persistently high beta and regional diversity. This outcome is in line with the hypothesis that the importance of community history increases with increasing isolation [9], [10]. However, since we tested only one possible initial species distribution, we cannot reliably confirm this hypothesis. Also, our results might have been influenced by the species' traits. The lasting effect of initial species composition was strongly contingent on the poor invasion ability of the superior competitor. Dispersal ability, competitive ability, disturbance tolerance and predator avoidance are further traits that have been suggested to potentially influence the importance of assembly history in structuring communities [9].

While the long-lasting effect of community history in weakly connected metacommunities led to persistently high beta and regional diversity, this was not the case for local diversity. The degree of connectivity affected only the temporal dynamics of local diversity, as abundances of superior competitors increased faster and abundances of inferior competitors declined faster the higher connectivity (Fig. 4, Fig. S2). Final local diversity, however, was unaffected by connectivity. Counterintuitively, failure of the superior competitor to reach dominance resulted in particularly low final local diversity (Fig. 5B), as the otherwise co-dominant species *Paramecium* reached very high relative abundances in these patches. Since both of the two alternative community states had low diversity, final average local diversity was low no matter if the metacommunity contained only one or both of these two community states. However, the decrease in diversity despite a release from the superior competitor could be a peculiarity of our model community. A number of other studies found that removal of a strong competitor can result in an increase in diversity [22]–[24]. Again, species' traits, competitive ability in particular, but also the specific sequences of community assembly will affect whether connectivity has a transitory or a persistent effect on local diversity. Since initial presence/absence of *Onychodromopsis* strongly influenced final local diversity of patches in weakly connected metacommunities, a different number of patches initially containing this species likely would have changed the result for local diversity.

While dynamics of local and regional diversity were strongly influenced by competition, dynamics of regional richness were driven by predation. Dispersal of the omnivorous species *Urostyla* resulted in extinction of its prey *Stylonychia* and thus a decline in regional richness. The time to extinction and hence the time to richness decline depended on the level of connectivity, with a faster decrease the higher the degree of connectivity (Fig. 2C). Competitive effects that resulted in persistent divergence of regional Shannon diversity did not affect regional richness since competition by *Onychodromopsis* resulted in considerably reduced abundances of inferior competitors but not in competitive exclusions. During the second half of the experiment, regional richness was thus unaffected by connectivity. Other metacommunity experiments with heterogeneously distributed predators found that dispersal of the predator resulted in a negative effect of connectivity on both local and regional prey species richness [8], [25]. In the present study, however, the positive effect of connectivity on local species richness through immigration of dispersal-limited species outweighed the negative effect of immigration of a predator. The low species number in our model community is a possible reason for the transitory effect of connectivity on local and regional richness. In natural systems with larger species pools and more complex and diverse species interactions, the likelihood for persistent effects of connectivity might be higher.

Our experimental setup combines elements from different metacommunity models. In correspondence with the patch dynamics perspective [1], the patches in our metacommunities were environmentally homogeneous and the dynamics were strongly influenced by a trade-off in competitive and colonization ability [26], [27]. However, the patch dynamics concept requires the creation of empty patches by stochastic or deterministic extinctions, which was not the case in our experiment. It was differences in community history, and hence absence of the superior competitor from some of the patches, that allowed the competition-colonization trade-off to operate. In correspondence with the mass effects perspective and source-sink models [1], [3], [4], we found decreasing beta and regional diversity with increasing connectivity as high dispersal resulted in homogenization of communities. However, the reason for persistent heterogeneity in community composition with low connectivity differed between source-sink models and our setup. It is environmental heterogeneity in source-sink models but initial differences in community composition in our experiment. The importance of spatial heterogeneity for an effect of connectivity on local, beta and regional diversity has been demonstrated by previous metacommunity experiments. Effects of connectivity on species richness and diversity were found to be stronger when community composition was heterogeneous rather than homogeneous, either through environmental heterogeneity [28], [29], through patchy disturbances [30], [31], or through differences in initial community composition [6]. Further, connectivity can affect species richness when heterogeneity in local community composition is created through stochastic colonizations or extinctions [25], [32]. Hence, different mechanisms can create persistent heterogeneity in weakly connected metacommunities, resulting in connectivity-diversity relationships similar to those predicted by source-sink models [4], [33].

While the mentioned metacommunity models only assume competitive interactions between species, an omnivorous species, *Urostyla*, was present in our communities. However, *Urostyla* quickly drove the one species it was able to feed upon to extinction in all three connectivity treatments. Thus, the omnivorous species affected the initial dynamics of regional richness but not the final outcome. A further difference between our experiment and many metacommunity models is that most models assume equally likely dispersal between all patches [4], [27], [34], which was not the case in our metacommunities. Patches within metacommunities differed in the number of tubing and were not connected with all other patches. However, species abundance data for the patches show that the spatial arrangement of patches had only minor effects on final abundances, though it did affect initial abundance patterns (Fig. S4). For example, *Paramecium* increased faster from zero in patches directly connected to patches with initial presence of *Paramecium* than in a patch without such a direct connection (Fig. S4 A–C). However, abundance values quickly converged to similar values in all six patches of a metacommunity. Likewise, the spatial arrangement of patches seemed to have only little effect on *Onychodromopsis*, the main driver of the diversity patterns. With low connectivity, *Onychodromopsis* did not build up populations in either of two patches in which it was not present initially, no matter whether the patch was connected to one or two other patches (Fig. S4D).

The second aim of our study was to analyse whether effects of connectivity on the ciliate community translated down to the resource community. While bacteria and flagellates were unaffected by connectivity, algal abundances declined with high and medium connectivity at the end of the experiment (Fig. 6). Although all of the five ciliate species consumed the algal resource, final abundances of the algae were (marginally) negatively correlated only with abundances of *Onychodromopsis*. A previous experiment showed that among the five ciliate species *Onychodromopsis* had by far the highest carrying capacity, at least twice as high as that of any of the other species [17]. Accordingly, total ciliate abundance in the present experiment was strongly positively correlated with abundance of *Onychodromopsis*. Ability of this species to build up very large populations likely resulted in strong depletion of the algal resource. Stronger dominance of the superior competitor with high and medium connectivity through a release from dispersal-limitation thus led to decreased algal abundances. Since the effect of connectivity on algal abundances appeared only at the very end of the experiment, we cannot exclude that resource patterns would converge over longer time scales. We are therefore not able to reliably answer the question of whether the effect of connectivity on the algal resource was transitory or persistent. Similar to our results, increased abundance of an efficient zooplanktonic grazer in highly connected metacommunities resulted in increased grazing pressure and altered phytoplankton community composition in a mesocosm experiment [15]. In contrast to our findings, however, Matthiessen and Hillebrand [5] found biomass production in a metacommunity of benthic microalgae to be maximized at intermediate connectivity. They suggested that highest local diversity at intermediate connectivity resulted in most efficient resource use and thus highest biomass production. Species' traits probably have a strong influence on how the effect of connectivity on diversity translates to the resource community. Also, in natural systems with a higher diversity of the resource community, diversity of the consumer community might be more important for efficient resource use than in our experiment.

In our study we showed that the effect of connectivity on diversity can be time-dependent and transitory, depending on the spatial scale and the measure of diversity. This result implies that repeated sampling can be necessary to reliably measure the importance of connectivity in structuring communities. However, despite our metacommunities being environmentally homogeneous, we found a time-independent and persistent effect of connectivity on beta and regional diversity. Greater importance of initial species composition with low connectivity resulted in reduced dominance of the superior competitor, an effect that translated down to the algal resource. The relative importance of individual species traits in determining outcomes in metacommunity experiments appears to be an interesting question for future studies.

## Supporting Information

Figure S1 **Local and regional diversity in the three connectivity treatments and an unconnected control.** The measures of diversity used were species richness (A, C) and the Shannon-Wiener index (B, D), computed at the local (A, B) and regional scale (C, D), respectively. Values are means ± SE, n = 3.
(TIF)

Figure S2 **Mean local abundances of the five ciliate species in the three connectivity treatments.** Abundances of (A) *Stylonychia*, (B) *Frontonia*, (C) *Onychodromopsis*, (D) *Paramecium*, and (E) *Urostyla* were log_10_-transformed and averaged over the six local communities of a metacommunity. Values are means ± SE, n = 3.
(TIF)

Figure S3 **Effect of initial presence/absence of** ***Onychodromopsis*** **on final relative abundances of the two dominant species.** Relative abundances of (A) *Onychodromopsis* and (B) *Paramecium* were averaged over local communities without initial presence of *Onychodromopsis* (black) and with initial presence of *Onychodromopsis* (white). Means ± SE (n_without_  = 6, n_with_  = 12).
(TIF)

Figure S4 **Spatially explicit dynamics.** Abundances of (A–C) *Paramecium*, (D–F) *Onychodromopsis*, (G–I) *Stylonychia*, (J–L) *Urostyla*, and (M–O) *Frontonia*, shown for each of the six communities of a metacommunity. Symbols depict the six initial species combinations (SO: closed circles, SOF: open circles, SOFPU: closed triangles, U: open triangles, SOFP: closed squares, FP: open squares; see Fig. 1 for species abbreviations and spatial arrangement of patches). Abundances were log_10_-transformed, values are means ± SE, n = 3.
(TIF)
