# Supplementary figures and images for: Transitory versus Persistent Effects of Connectivity in Environmentally Homogeneous Metacommunities
Source: PLoS One. 2012 Aug 30;7(8):e44555. doi: 10.1371/journal.pone.0044555 (PMC3431365; doi:10.1371/journal.pone.0044555)

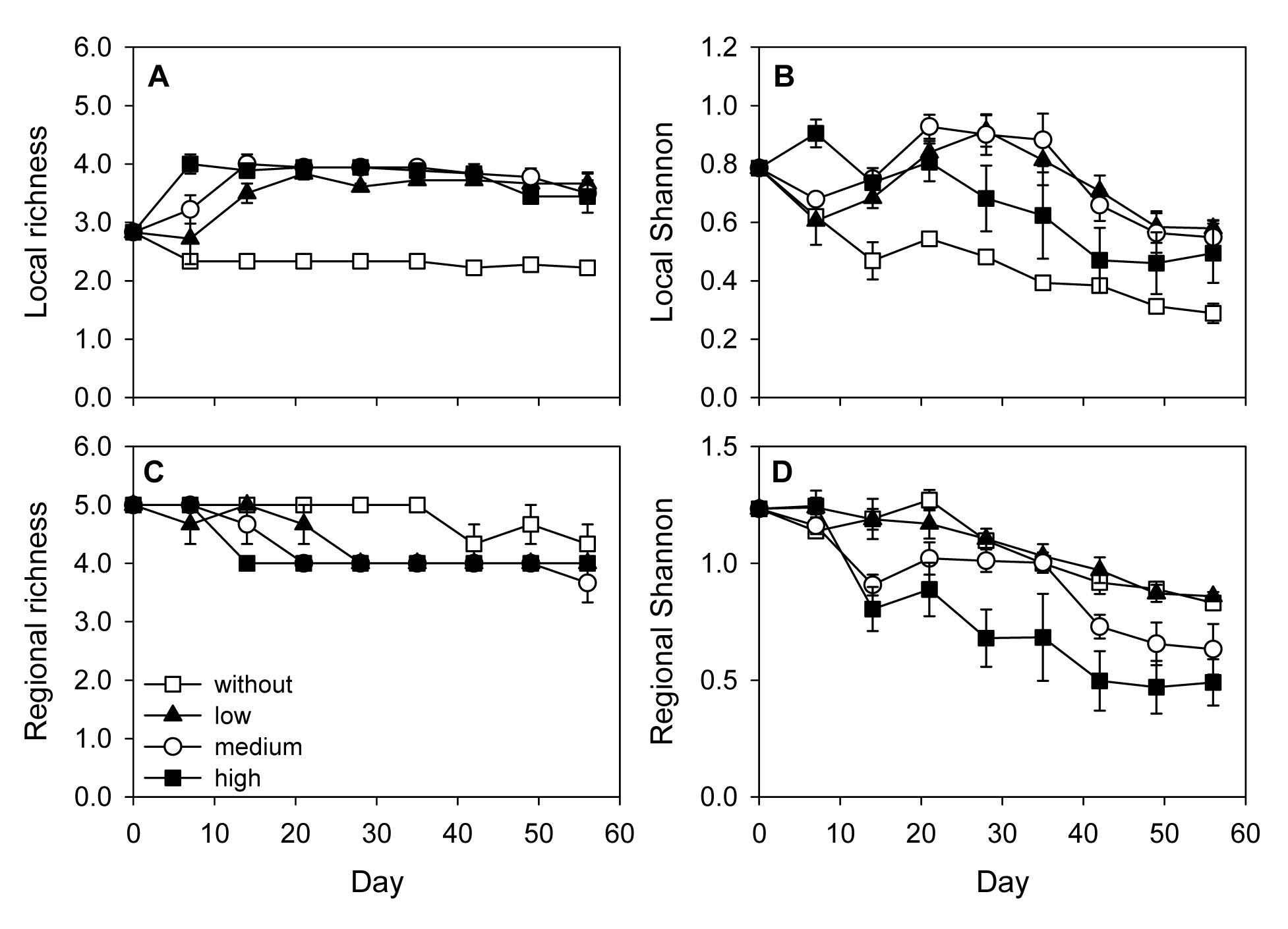

Supplement: Figure S1 — Local and regional diversity in the three connectivity treatments and an unconnected control. The measures of diversity used were species richness (A, C) and the Shannon-Wiener index (B, D), computed at the local (A, B) and regional scale (C, D), respectively. Values are means ± SE, n = 3. (TIF) [file pone.0044555.s001.tif]

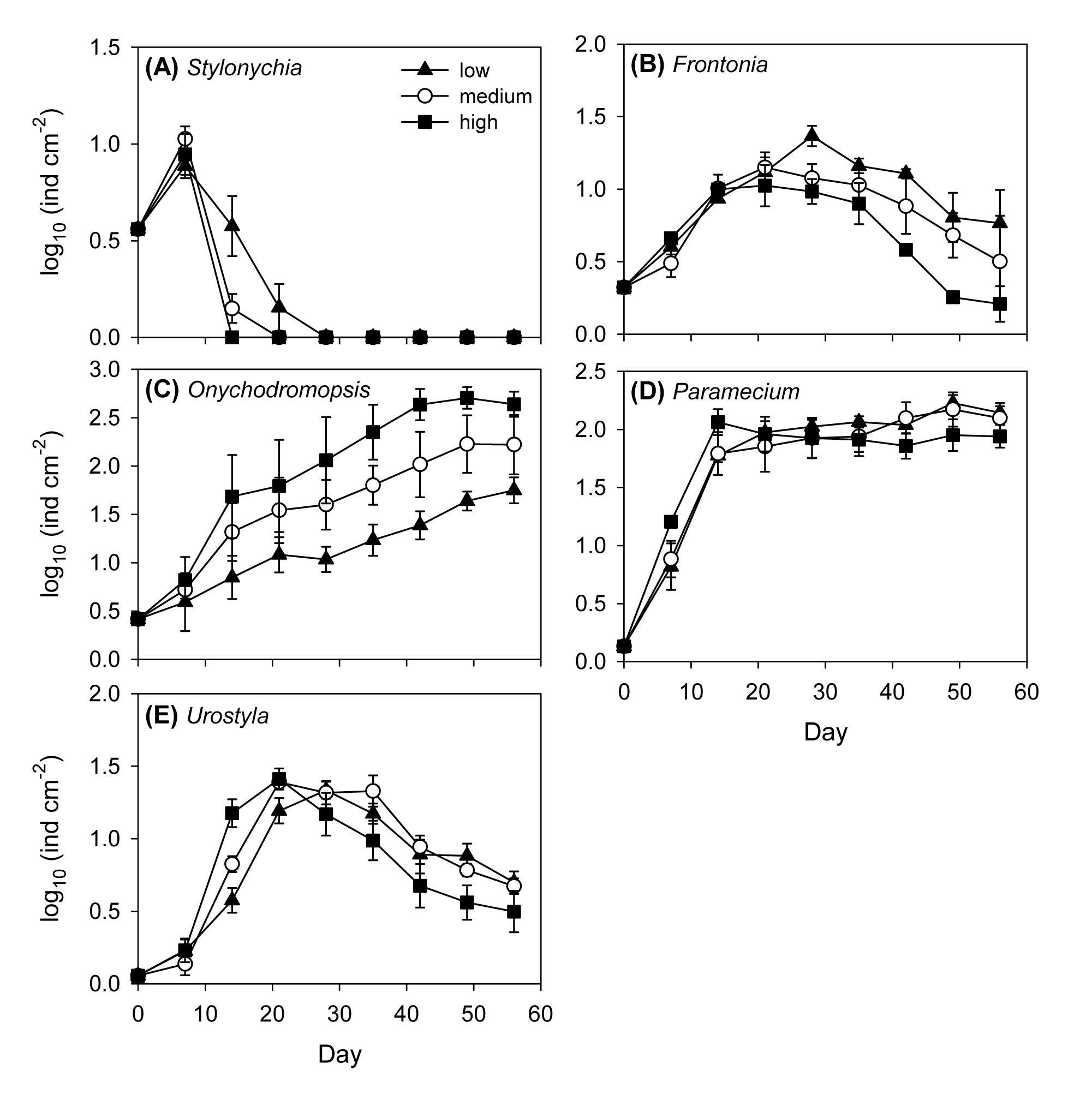

Supplement: Figure S2 — Mean local abundances of the five ciliate species in the three connectivity treatments. Abundances of (A) Stylonychia, (B) Frontonia, (C) Onychodromopsis, (D) Paramecium, and (E) Urostyla were log10-transformed and averaged over the six local communities of a metacommunity. Values are means ± SE, n = 3. (TIF) [file pone.0044555.s002.tif]

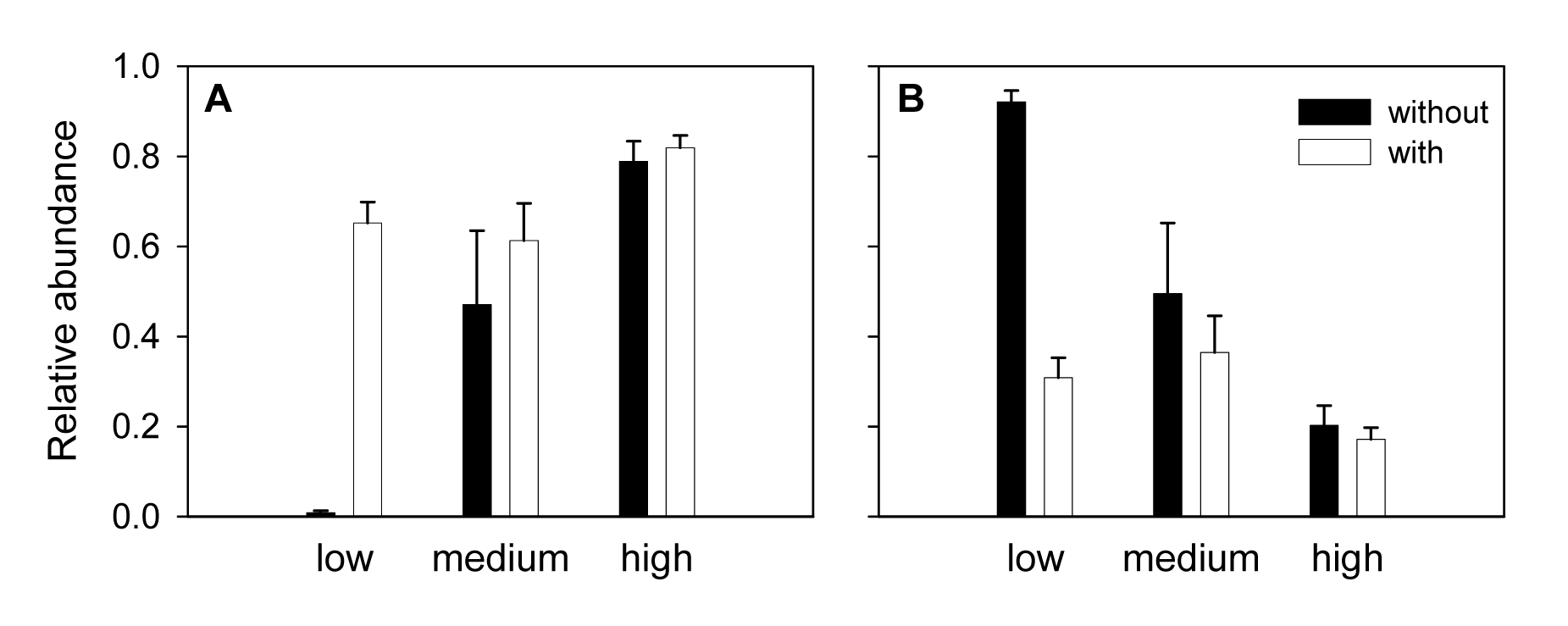

Supplement: Figure S3 — Effect of initial presence/absence of Onychodromopsis on final relative abundances of the two dominant species. Relative abundances of (A) Onychodromopsis and (B) Paramecium were averaged over local communities without initial presence of Onychodromopsis (black) and with initial presence of Onychodromopsis (white). Means ± SE (nwithout = 6, nwith = 12). (TIF) [file pone.0044555.s003.tif]

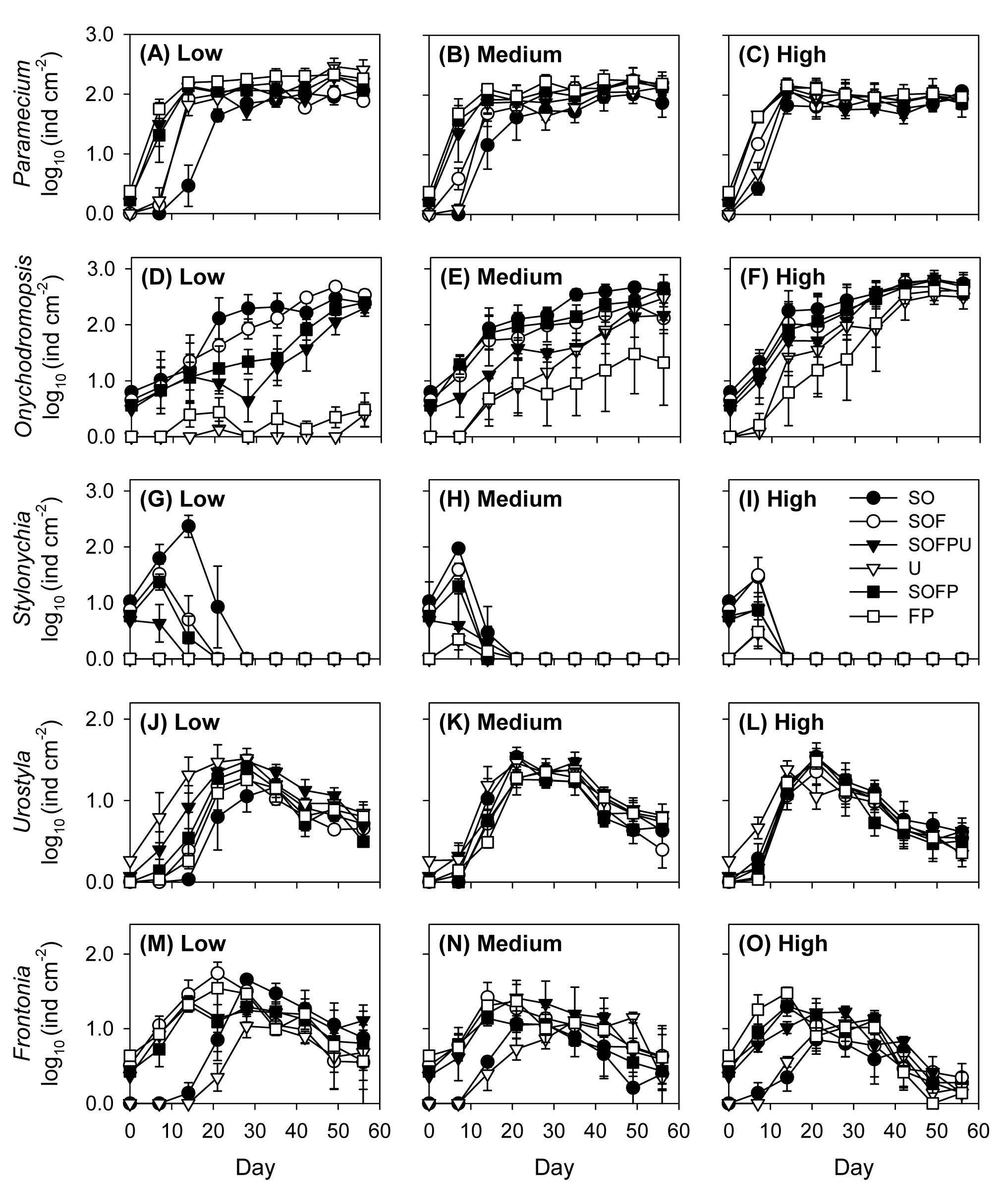

Supplement: Figure S4 — Spatially explicit dynamics. Abundances of (A–C) Paramecium, (D–F) Onychodromopsis, (G–I) Stylonychia, (J–L) Urostyla, and (M–O) Frontonia, shown for each of the six communities of a metacommunity. Symbols depict the six initial species combinations (SO: closed circles, SOF: open circles, SOFPU: closed triangles, U: open triangles, SOFP: closed squares, FP: open squares; see Fig. 1 for species abbreviations and spatial arrangement of patches). Abundances were log10-transformed, values are means ± SE, n = 3. (TIF) [file pone.0044555.s004.tif]
